# Complete genome sequence of the nitrogen-fixing bacterium *Azospirillum humicireducens* type strain SgZ-5^T^

**DOI:** 10.1186/s40793-018-0322-2

**Published:** 2018-10-16

**Authors:** Zhen Yu, Guiqin Yang, Xiaoming Liu, Yueqiang Wang, Li Zhuang, Shungui Zhou

**Affiliations:** 1 Guangdong Key Laboratory of Agricultural Environment Pollution Integrated Control, Guangdong Institute of Eco-Environmental Science & Technology, Guangzhou, 510650 China; 2 School of Environment, Guangdong Key Laboratory of Environmental Pollution and Health, Jinan University, Guangzhou, 510632 China; 3 Fujian Provincial Key Laboratory of Soil Environmental Health and Regulation, College of Resources and Environment, Fujian Agriculture and Forestry University, Fuzhou, 350002 China

**Keywords:** Azospirillum humicireducens, Complete genome, Nitrogen fixation, PGPP

## Abstract

The *Azospirillum humicireducens* strain SgZ-5^T^, belonging to the Order *Rhodospirillales* and the Family *Rhodospirillaceae*, was isolated from a microbial fuel cell inoculated with paddy soil. A previous work has shown that strain SgZ-5^T^ was able to fix atmospheric nitrogen involved in plant growth promotion. Here we present the complete genome of *A. humicireducens* SgZ-5^T^, which consists of a circular chromosome and six plasmids with the total genome size of 6,834,379 bp and the average GC content of 67.55%. Genome annotations predicted 5969 protein coding and 85 RNA genes including 14 rRNA and 67 tRNA genes. By genomic analysis, we identified a complete set of genes that is potentially involved in nitrogen fixation and its regulation. This genome also harbors numerous genes that are likely responsible for phytohormones production. We anticipate that the *A. humicireducens* SgZ-5^T^ genome will contribute insights into plant growth promoting properties of *Azospirillum* strains.

**Electronic supplementary material:**

The online version of this article (10.1186/s40793-018-0322-2) contains supplementary material, which is available to authorized users.

## Introduction

*Bacteria* that live in the plant rhizosphere and possess a large array of potential mechanisms to enhance plant growth are considered as PGPR [1–3]. *Azospirillum* represents a well characterized genus of PGPR due to its capacity of fixing atmospheric nitrogen [4, 5]. Although the exact contribution of *Azospirillum* to biological nitrogen fixation in plant growth promotion is debated [2], agricultural applications of the genus *Azospirillum* have been still developed [6, 7]. Another main characteristic of *Azospirillum* proposed to explain plant growth promotion has been related to its ability to produce phytohormones [8, 9].

At present, there are 17 species within the genus *Azospirillum* [10], of which the nitrogen-fixing bacterium *A. humicireducens* SgZ-5^T^, the focus species of this study, was initially isolated from the anode biofilm of a MFC. A soil sample collected from paddy field in Guangzhou City, Guangdong Province, China (23.18^o^ N 113.36^o^ E) was used as inoculating source of the MFC. In a previous report [11], the nitrogen-fixing capability of strain SgZ-5^T^ was confirmed by acetylene-reduction assay and identification of a *nifH* gene. Furthermore, this strain has the ability to grow under anaerobic conditions via the oxidation of various organic compounds coupled to the reduction of humus [11], showing its potential use in plant rhizosphere. Here, we describe the physiological features together with the whole genome sequence of *A. humicireducens* SgZ-5^T^.

## Organism information

### Classification and features

**Fig. 1:**
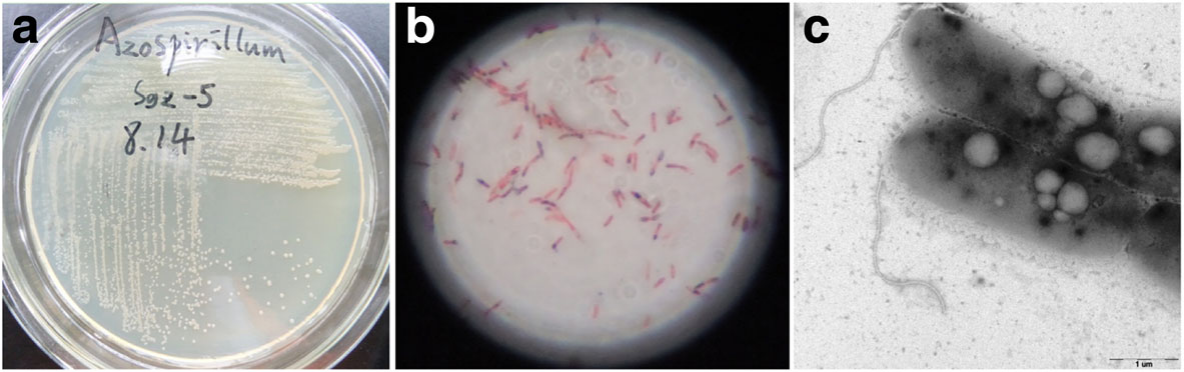
Images of the *A. humicireducens* SgZ-5^T^. **a** Colonies of the strain on NA agar plate, **b** light microscopy and **c** transmission electron microscopy of the strain

*A. humicireducens* SgZ-5^T^ is a Gram-negative, facultative anaerobic, motile, spiral, straight to slightly curved rod-shaped bacterium (Fig. 1), belonging to the Order *Rhodospirillales* and the Family *Rhodospirillaceae*. The strain grew optimally in the conditions of 30 °C, pH 7.2, and 1% NaCl [11]. On NA, strain SgZ-5^T^ formed cream-colored, round, smooth, convex and non-translucent colonies (Fig. 1). With AQDS as the sole terminal electron acceptor, strain SgZ-5^T^ could utilize pyruvate, glucose and acetate as electron donors under anaerobic conditions [11]. Strain SgZ-5^T^ was able to use a range of carbon substrates including *N*-Acetyl-glucosamine, citrate, D-ribose, meso-inositol, D-saccharose, D-maltose, L-rhamnose, suberic acid, malonate, acetate, L-serine, salicin, L-lactate, L-alanine, gluconate, 2-keto-gluconate, glycogen, D-mannitol, D-glucose, D-melibiose, L-fucose, D-sorbierite, L-arabinose, L-histidine, 3-hydroxy-butyric acid, 4-hydroxy-benzoic acid, L-proline, capric acid, adipic acid and malic acid [11] (Table 1).

**Table 1:** Classification and general features of *A. humicireducens* SgZ-5^T^ according to the MIGS recommendations [16]

MIGS ID	Property	Term	Evidence code^a^
	Current Classification	Domain *Bacteria*	TSA [22]
		Phylum *Proteobacteria*	TSA [33]
		Class *Alphaproteobacteria*	TSA [34]
		Order *Rhodospirillales*	TSA [35, 36]
		Family *Rhodospirillaceae*	TSA [35, 36]
		Genus *Azospirillum*	TSA [37, 38]
		Species *Azospirillum humicireducens*	TSA [11]
		Type strain SgZ-5=CCTCC AB 2012021=KACC 16605	TSA [11]
	Gram stain	Negative	TSA [11]
	Cell shape	Spiral, straight to slightly curved rods	TSA [11]
	Motility	Motile	TSA [11]
	Sporulation	Nonsporulating	NSA
	Temperature range	25–37 °C	TSA [11]
	Optimum temperature	30 °C	TSA [11]
	pH range; Optimum	5.5–8.5; 7.2	TSA [11]
	Carbon source	Acetate, L-lactate, citrate, D-ribose, L-rhamnose, D-glucose, N-Acetyl-glucosamine, meso-inositol, D-saccharose, D-maltose, suberic acid, malonate, L-serine, salicin, L-alanine, gluconate, glycogen, 2-keto-gluconate, D-mannitol, D-melibiose, L-fucose, D-sorbierite, L-arabinose, L-histidine, 3-hydroxy-butyric acid, 4-hydroxy-benzoic acid, L-proline, capric acid, adipic acid and malic acid	TSA [11]
MIGS-6	Habitat	Paddy soil	TSA [11]
MIGS-6.3	Salinity	NaCl 0–1% (*w/v*)	TSA [11]
MIGS-22	Oxygen requirement	Facultative anaerobic	TSA [11]
MIGS-15	Biotic relationship	Free living	NAS
MIGS-14	Pathogenicity	Not reported	
MIGS-4	Geographic location	Guangzhou City, Guangdong Province, China	NAS
MIGS-5	Sample collection time	Dec 2011	NAS
MIGS-4.1	Latitude	23.18^o^ N	NAS
MIGS-4.2	Longitude	113.36^o^ E	NAS
MIGS-4.3	Depth	0.1 m beneath the surface	NAS
MIGS-4.4	Altitude	40 m	NAS

^a^Evidence code – *IDA* Inferred from direct assay, *TAS* Traceable author statement (i.e., a direct report exists in the literature), *NAS* Non-traceable author statement (i.e., not directly observed for the living, isolated sample, but based on a generally accepted property for the species, or anecdotal evidence). These evidence codes are from the Gene Ontology Project [39]

**Fig. 2:**
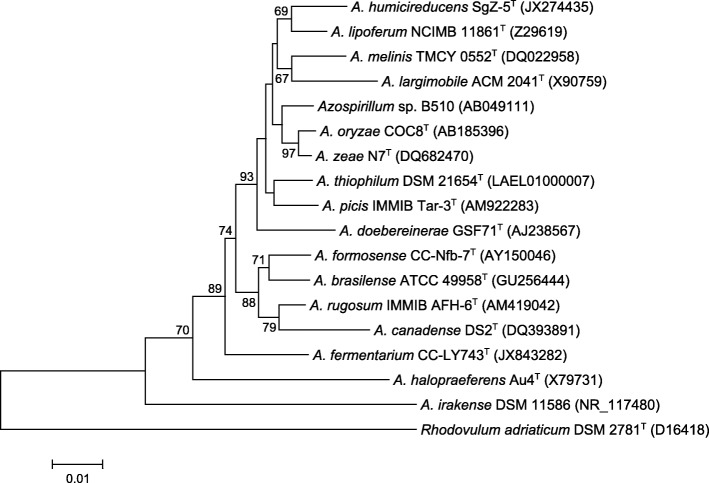
Phylogenetic tree highlighting the position of *A. humicireducens* SgZ-5^T^ relative to other type strains within the genus *Azospirillum*. The strains and their corresponding GenBank accession numbers of 16S rRNA genes were indicated in parentheses. The sequences were aligned using Clustal W and the neighbor-joining tree was constructed based on kimura 2-paramenter distance model by using MEGA 5. Bootstrap values above 50 % were obtained from 1000 bootstrap replications. *Bar*, 0.01 substitutions per nucleotide position. *Rhodovulum adriaticum* DSM 2781^T^ was used as an outgroup

A phylogenetic tree was constructed from aligning the 16S rRNA gene sequences of strain SgZ-5^T^ and type strains of the genus *Azospirillum* by MEGA 5 using the neighbour-joining method [12]. The phylogenetic position of strain SgZ-5^T^ is shown in Fig. 2, where *A. humicireducens* can be grouped as a *Azospirillum* species, forms a distinct subclade together with *A. lipoferum* that are known as a biofertilizer widely used for agricultural production [13, 14]. The 16S rRNA gene of strain SgZ-5^T^ is 98% similar to that of *A. lipoferum* NCIMB 11861^T^. Since *nifH* gene is highly conserved among nitrogen-fixing *Proteobacteria* [15], a *nifH*-based phylogenetic tree was constructed to identify the relationship of *A. humicireducens* to other species within the genus *Azospirillum* and related genus (Additional file 1). The phylogenetic reconstruction indicated the close relationship of the *A. humicireducens* SgZ-5^T^ *nifH* gene with that from *Azospirillum* sp. B510.

## Genome sequencing information

### Genome project history

*A. humicireducens* SgZ-5^T^ was selected for genome sequencing on the basis of its biotechnological potential in agricultural applications as a PGPR likely harboring multiple PGPP [11]. The complete genome sequences have been deposited at Gen-Bank/EMBL/DDBJ under the accession numbers CP015285.1, CP028902-CP028907. Project information is available from Genome Online database number Gp0150267 at Joint Genome Institute. In Table 2, we summarize the project information and its association with Minimum Information about a Genome Sequence (MIGS) [16].

**Table 2:** Genome sequencing project information

MIGS ID	Property	Term
MIGS-31	Finishing quality	Complete
MIGS-28	Libraries used	Three libraries (a paired-end library and two mate-pair libraries)
MIGS-29	Sequencing platforms	Illumina Hiseq 2500
MIGS-31.2	Fold coverage	259×
MIGS-30	Assemblers	SOAP*denovo* 2.04 [17]
MIGS-32	Gene calling method	GeneMarkS+ [18]
	Locus Tag	A6A40
	Genbank ID	CP015285.1, CP028902-CP028907
	Genbank Date of Release	April 18, 2018
	GOLD ID	Gp0150267
	Bioproject	PRJNA318554
MIGS-13	Source Material Identifier	SgZ-5^T^
	Project relevance	Type strain, nitrogen fixation, plant growth promotion

### Growth conditions and genomic DNA preparation

*A. humicireducens* SgZ-5^T^ was routinely cultured in NB medium containing (L^− 1^) 5 g peptone, 3 g beef extract and 5 g NaCl at 30 °C. For genome sequencing, total genomic DNA was extracted from 10 mL overnight cultures using a DNA extraction kit following the manufacture’s instructions (Aidlab). Quantification and quality control of the genomic DNA were completed by using a Qubit fluorometer (Invitrogen, CA, USA) with Qubit dsDNA BR Assay kit and 0.7% agarose gel electrophoresis with λ-Hind III digest DNA marker.

### Genome sequencing and assembly

Complete genome sequencing was performed on an Illumina HiSeq 2500 system by constructing three DNA libraries (a paired-end library with insert size of 491 bp, and two mate pair libraries with insert sizes of 2.5 and 6.9 kb). After filtering low quality and Illumina PCR adapter reads, a total of 1967 Mb clean data were obtained from 2052 Mb raw data. Subsequently, all reads data were *denovo* assembled into a circular contig with 259 folds of genomic coverage, using SOAPdenovo v.2.04 [17]. Detailed genome sequencing project information is shown in Table 2.

### Genome annotation

Gene prediction was carried out by GeneMarkS v.4.6 [18]. Function annotation of predicted ORFs was performed based on a BLASTP search against NCBI nonredundant protein database and COG database. Transfer RNAs, rRNAs and sRNA were predicted using tRNAscan-SE v.1.31 with the bacterial model, RNAmmer v.1.2 and Rfam database v.9.1, respectively [19–21]. The CRISPRs were identified by using the CRISPR database [22]. The prediction of genes with signal peptides and transmembrane helices were performed by SignalP server v.4.1 [23] and TMHMM server v.2.0 [24], respectively. The secondary metabolism gene cluster was predicted according to the antiSMASH v.3.0 procedure [25].

## Genome properties

**Fig. 3:**
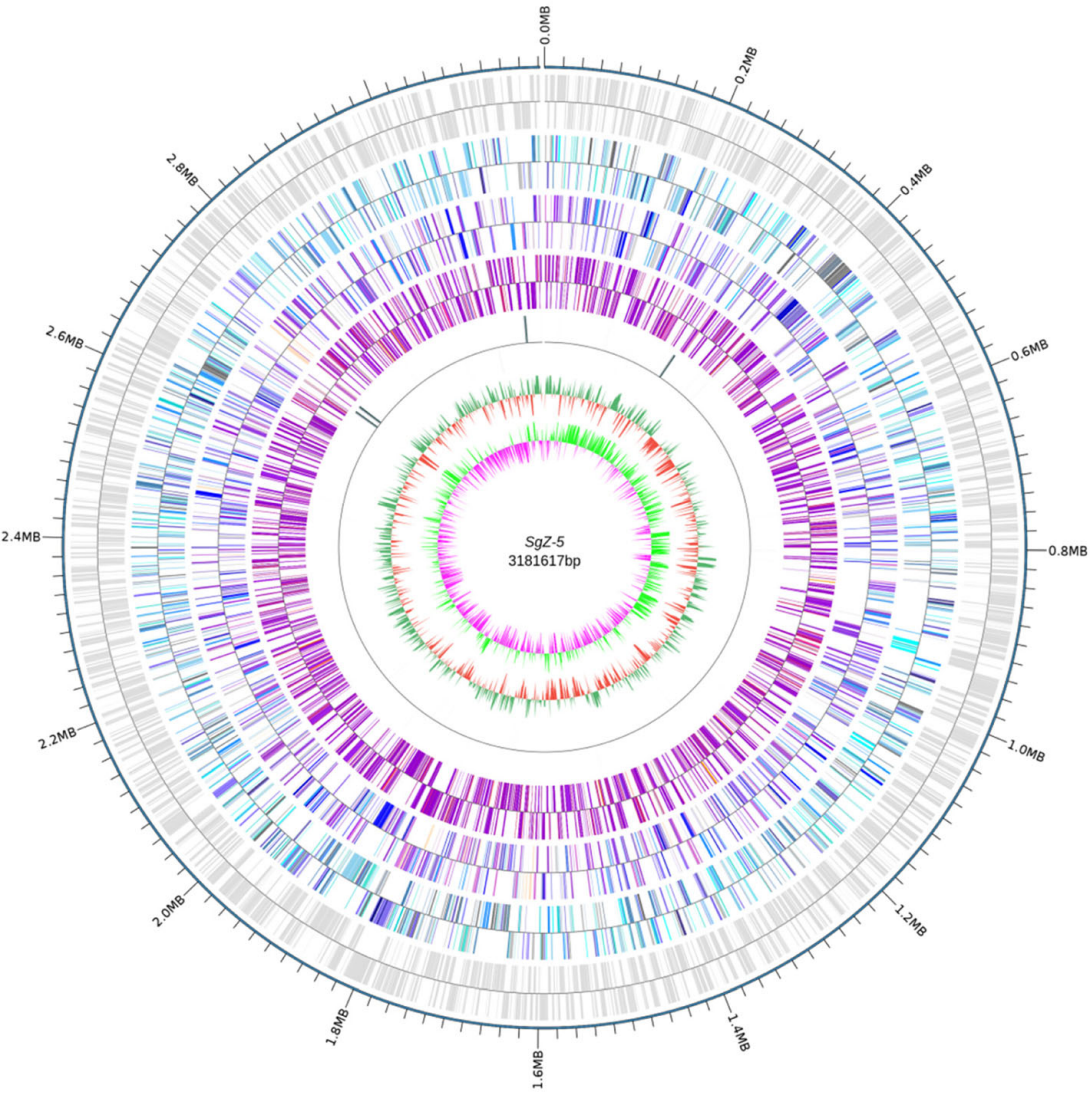
Circular map of the chromosome of *A. humicireducens* SgZ-5^T^. From center to outside, circle 1 illustrates the GC skew. Circle 2 shows GC content (peaks out/inside the circle indicate values higher or lower than the average G+C content, respectively). Circle 3 denotes ncRNA genes. Circles 4, 5 and 6 indicate the CDSs, colored according to COG, KEGG and GO categories, respectively. Circle 7 demonstrates the predicted protein-coding sequences

The genome of *A. humicireducens* SgZ-5^T^ comprises a circular chromosome of 3,181,617 bp and six circular plasmids, designated as pYZ1 (715,112 bp), pYZ2 (1,008,603 bp), pYZ3 (252,411 bp), pYZ4 (338,445 bp), pYZ5 (626,509 bp) and pYZ6 (711,682 bp) (Table 3). The total size of the genome is 6,834,379 bp, and the average GC content is 67.55%. The genome contains 6054 genes with the total length of 5,902,731 bp, of which 5969 (98.6%) are protein coding genes. There are 85 RNA genes (1.4%), including 14 rRNA and 67 tRNA genes. A total of 4844 genes (80.0%) have been assigned a predicted function while the rest have been designated as hypothetical proteins. Genome statistics are summarized in Table 4 and a graphical map is represented in Fig. 3. Furthermore, 4550 (75.2%) genes were assigned to 21 COG functional categories. The distribution of genes into different COG functional categories is provided in Table 5. Six *Azospirillum* species genomes (including *A. humicireducens*) of characterized strains are compared in Table 6. Almost all of these *Azospirillum* genomes consisting of 6–8 replicons have the total size of 6.5–7.6 Mb and the average GC content of 67.5–70.7%, and contain the total genes in the range of 5951 to 6982 [3, 6, 26, 27]. Furthermore, the main features of *A. humicireducens* SgZ-5^T^ genome are close to those of *A. lipoferum* 4B genome.

**Table 3:** Summary of genome: one chromosome and six plasmids

Label	Size (bp)	Topology	INSDC identifier	RefSeq ID
Chromosome	3,181,617	Circular	CP015285.1	NZ_CP015285.1
pYZ1	715,112	Circular	CP028902.1	NA
pYZ2	1,008,603	Circular	CP028903.1	NA
pYZ3	252,411	Circular	CP028904.1	NA
pYZ4	338,445	Circular	CP028905.1	NA
pYZ5	626,509	Circular	CP028906.1	NA
pYZ6	711,682	Circular	CP028907.1	NA

**Table 4:** Genome statistics of *A. humicireducens* SgZ-5^T^

Attribute	Genome (total)	
	Value	% of total
Genome size (bp)	6,834,379	100.00
DNA coding (bp)	5,902,731	86.37
DNA G + C (bp)	4,616,422	67.55
DNA scaffolds	7	
Total genes	6054	100.00
Protein coding genes	5969	98.60
RNA genes	85	1.40
rRNA genes	14	0.23
tRNA genes	67	1.11
Pseudo genes	194	3.20
Genes in internal clusters	NA	
Genes with function prediction	4844	80.01
Genes assigned to COGs	4550	75.16
Genes with signal peptides	425	7.02
Genes with transmembrane helices	1022	16.88
CRISPR repeats	3

**Table 5:** Number of genes associated with general COG functional categories

Code	Value	% of total^a^	Description
J	182	2.98	Translation, ribosomal structure and biogenesis
A	0	0.00	RNA processing and modification
K	357	5.85	Transcription
L	175	2.87	Replication, recombination and repair
B	1	0.02	Chromatin structure and dynamics
D	38	0.62	Cell cycle control, cell division, chromosome partitioning
V	80	1.31	Defense mechanisms
T	338	5.54	Signal transduction mechanisms
M	218	3.57	Cell wall/membrane/envelope biogenesis
N	73	1.20	Cell motility
U	58	0.95	Intracellular trafficking, secretion, and vesicular transport
O	162	2.65	Posttranslational modification, protein turnover, chaperones
C	342	5.60	Energy production and conversion
G	263	4.31	Carbohydrate transport and metabolism
E	448	7.34	Amino acid transport and metabolism
F	81	1.33	Nucleotide transport and metabolism
H	160	2.62	Coenzyme transport and metabolism
I	139	2.28	Lipid transport and metabolism
P	333	5.45	Inorganic ion transport and metabolism
Q	144	2.36	Secondary metabolites biosynthesis, transport and catabolism
R	227	3.72	General function prediction only
S	731	11.97	Function unknown
–	1555	25.47	Not in COGs

^a^The total is based on the total number of protein coding genes in the annotated genome

**Table 6:** Genome statistics comparison among characterized *Azospirillum* species^a^

Genome name	1			2	3	4	5	6
	Sp 7	Az39	Sp245	4B	DSM 3675	BV-S	B510	SgZ-5
Genome size (Mb)	6.6	7.4	7.5	6.8	6.5	7.6	7.6	6.8
Plasmids	5	5	6	6	NA	7	6	6
G + C content (%)	68.3	68.5	68.5	67.7	70.7	68.2	67.6	67.5
Total genes	5951	6713	6982	6137	5999	6684	6692	6054
Protein coding genes	5828	6596	6882	6027	5925	6575	6582	5969
RNA genes	123	117	100	110	74	109	110	85
rRNA genes	29	26	15	26	11	26	26	14
tRNA genes	90	87	81	80	59	79	80	67
Pseudogenes	98	188	375	77	128	126	195	194
Frameshifted genes	38	61	286	21	27	53	85	67
CRISPR	2	NA	NA	3	4	NA	5	3

^a^the *Azospirillum* species are numbered as: 1, *A. brasilense* [26, 27]; 2, *A. lipoferum* [26]; 3, *A. halopraeferens* (RefSeq ID: NZ_AUCF00000000.1); 4, *A. thiophilum* [3]; 5, *Azospirillum* sp. [6]; 6, *A. humicireducens*

## Insights into the genome sequence

Nitrogen fixation is the major proposed mechanism, by which *Azospirillum* affects plant growth [2, 4]. A complete set of genes encoding enzymes involved in nitrogen fixation was found in the genomic analysis of *A. humicireducens* SgZ-5^T^ (Table 7). The main genes involved in this process are *nif* genes, of which *nifDK* genes (A6A40_02900 and A6A40_02895) annotated as nitrogenase molybdenum-iron proteins and *nifH* gene (A6A40_02905) encoding dinitrogenase reductase protein have been identified. In the upstream region of the *nifHDK* operon, we have found that *nifEN* genes (A6A40_02875 and A6A40_02870) involved in synthesis of the molybdenum-iron cofactor of nitrogenase are clustered into a single operon together with *nifX* (A6A40_02865). Furthermore, the genome of *A. humicireducens* SgZ-5^T^ has *nifUSVW* genes (A6A40_02235, A6A40_02230, A6A40_02225 and A6A40_02215), which are separated from the structural *nifENX* operon by about 160 kb.

**Table 7:** Genes of *A. humicireducens* SgZ-5^T^ involved in nitrogen fixation

Locus Tag	Size/aa	Gene	Gene product
A6A40_02185	852	*fixA*	Electron transfer flavoprotein beta subunit
A6A40_02190	1080	*fixB*	Electron transfer flavoprotein alpha chain
A6A40_02195	1302	*fixC*	Flavoprotein-ubiquinone oxidoreductase
A6A40_09085	210	*fixU*	Nitrogen fixation protein
A6A40_02200	285	*fixX*	Ferredoxin-like protein
A6A40_09040	1866	*nifA*	Nif-specific transcriptional activator
A6A40_09050	1518	*nifB*	Nitrogenase FeMo cofactor biosynthesis protein
A6A40_02900	1440	*nifD*	Nitrogenase molybdenum-iron protein alpha chain
A6A40_02875	1407	*nifE*	Nitrogenase molybdenum-cofactor biosynthesis protein
A6A40_02905	897	*nifH*	Nitrogenase iron protein
A6A40_02895	1560	*nifK*	Nitrogenase molybdenum-iron protein subunit beta
A6A40_02870	1371	*nifN*	Nitrogenase molybdenum-cofactor biosynthesis protein
A6A40_02230	1206	*nifS*	Nitrogenase metalloclusters biosynthesis protein
A6A40_02235	924	*nifU*	Iron-sulfur cluster assembly scaffold protein
A6A40_02225	1122	*nifV*	Homocitrate synthase
A6A40_02215	336	*nifW*	Nitrogenase-stabilizing/protective protein
A6A40_02865	399	*nifX*	Nitrogenase molybdenum-iron protein
A6A40_09070	333	*nifZ*	Nitrogenase P-cluster assembly
A6A40_09075	306	*nifZ*	Nitrogenase P-cluster assembly
A6A40_02220	852	*cysE*	Serine acetyltransferase
A6A40_02925	909	*draG*	ADP-ribosyl-[dinitrogen reductase] hydrolase
A6A40_02920	891	*draT*	ADP-ribosyl-[dinitrogenase reductase] transferase
A6A40_07245	2847	*glnD*	[Protein-PII] uridylyltransferase
A6A40_07685	339	*glnB*	Nitrogen regulatory protein P-II
A6A40_05220	1200	*ntrB*	Nitrogen regulation sensor histidine kinase
A6A40_05215	1146	*ntrC*	Nitrogen regulation response regulator
A6A40_05205	1401	*ntrX*	Sigma-54-dependent transcriptional regulator
A6A40_05210	2319	*ntrY*	Nitrogen regulation sensor histidine kinase

**Fig. 4:**
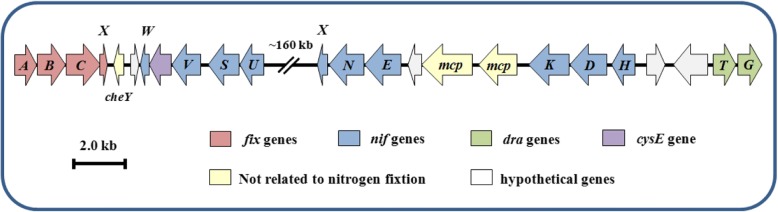
Organization of the nitrogen fixation gene cluster in *A. humicireducens* SgZ-5^T^. Arrows represent genes and their respective direction of transcription. Genes are colored as depicted in the lower box

Organization of the nitrogen fixation gene cluster in *A. humicireducens* SgZ-5^T^ is presented in Fig. 4. Except for the separately transcribed *nifA* (A6A40_09040), *nifB* (A6A40_09050) and *nifZ* genes (A6A40_09070 and A6A40_09075), all the *nif* genes have resided in the nitrogen fixation gene cluster of 176.7 kb. Besides, an operon containing *fixABCX* genes (A6A40_02185, A6A40_02190, A6A40_02195 and A6A40_02220) responsible for electron transfer to nitrogenase is located upstream of this gene cluster. Nevertheless, the *fixABCX* operon is generally regulated by a transcriptional activator NifA protein for all nitrogen-fixing bacteria in the genus *Azospirillum* studied so far [5]. Furthermore, *draTG* genes (A6A40_02920 and A6A40_02925) implicated in posttranslational regulatory process of nitrogenase activity were found in the downstream of and divergently oriented with respect to *nifHDK* genes. On the whole, the nitrogen fixation gene cluster of *A. humicireducens* SgZ-5^T^ was in agreement with that in *A. brasilense*, *A. lipoferum* and *Azospirillum* sp. B510 [6, 26, 28, 29], suggesting that nitrogen fixation process demands the systematic action of various genes.

Since tryptophan is a main precursor for biosynthesis of IAA, a well-known phytohormone [30], the genes in *A. humicireducens* SgZ-5^T^ related to the production of this amino acid have been analyzed (Additional file 2). The genome harbors three genes *trpE*, *trpG* and *trpEG* (A6A40_04380, A6A40_04655 and A6A40_05775), each encoding the key enzyme anthranilate synthase in tryptophan biosynthesis. Together with *trpG*, the genes *trpD* (A6A40_04650) and *trpC* (A6A40_04645) form a gene cluster of 2.4 kb. Except for anthranilate synthase, this *trpGDC* gene cluster encodes anthranilate phosphoribosyltransferase and indole-3-glycerol phosphate synthase, which plays a role in synthesis of tryptophan used in multiple biological processes including IAA biosynthesis [31]. The same *trpGDC* cluster was previously found in *A. brasilense* [32]. Although the *ipdC* gene, related to the indole-3-pyruvate pathway for the biosynthesis of IAA [30], was not discovered in the *A. humicireducens* SgZ-5^T^ genome, alternative pathway might exist in SgZ-5^T^. In the genome, A6A40_22745 and A6A40_22755 were assigned as candidates for *iaaM* and *iaaH* genes, respectively. These two genes were also found in the *Azospirillum* sp. B510 genome, and are known to be involved in the IAM pathway for IAA biosynthesis by catalyzing the decarboxylation of tryptophan into IAM and the hydrolysis of IAM to produce IAA [6, 30].

The *A. humicireducens* SgZ-5^T^ genome also contains a *terpene* gene cluster of 24.0 kb consisting of 23 genes (A6A40_04945, A6A40_04950, A6A40_04955, …, A6A40_05055) (Additional file 3). This gene cluster encodes a series of proteins, which are involved in the biosynthesis of secondary metabolite production of terpenoid. Thereinto, A6A40_05010 was indentified as the *crtB* gene, encoding phytoene synthase involved in the biosynthesis of carotenoid. Similar genes in this gene cluster were previously observed in the *A. lipoferum* 4B genome [7, 26]. Furthermore, some phytohormones including gibberellins and abscisic acid with over 120 types found in plants, fungi, and bacteria, are synthesized through the terpenoid pathway [2]. Therefore, *A. humicireducens* SgZ-5 exhibits an attractive application as a PGPR likely harboring multiple PGPP in agriculture.

## Conclusion

We report here an inventory of the genomic features of the nitrogen-fixing bacterium *A. humicireducens* SgZ-5^T^. The genome sequence of strain SgZ-5^T^ revealed further genetic elements involved in nitrogen fixation and its regulation, as well as in the production of phytohormones. We anticipate that knowledge of this genome will contribute to new insights into the mechanisms of plant growth stimulation through genomic comparisons among available complete genomes of *Azospirillum* strains.

## Additional files

Additional file 1: Phylogenetic tree based on the partial *nifH* gene sequences showing the position of *A. humicireducens* SgZ-5^T^ relative to other species within the genus *Azospirillum* and related genus. The strains and their corresponding GenBank accession numbers of *nifH* gene were indicated in parentheses. The sequences were aligned using Clustal W and the neighbor-joining tree was constructed based on kimura 2-paramenter distance model by using MEGA 5. Bootstrap values above 50% were obtained from 1000 bootstrap replications. *Bar*, 0.01 substitutions per nucleotide position. *Leptospirillum ferriphilum* YSKT was used as an outgroup. (DOCX 64 kb)

Additional file 2: Genes of *A. humicireducens* SgZ-5^T^ involved in biosynthesis of tryptophan. (DOCX 16 kb)

Additional file 3: Genes of *A. humicireducens* SgZ-5^T^ located in a terpene gene cluster. (DOCX 16 kb)

## Abbreviations

AQDS: Anthraquinone-2, 6-disulfonate
IAA: Indole-3-acetic acid
IAM: Indole-3-actamide
MFC: Microbial fuel cell
NA: Nutrient Agar
NB: Nutrient Broth
PGPP: Plant growth promoting properties
PGPR: Plant growth-promoting rhizobacteria
