# Supplementary material for: Complete genome sequence of the nitrogen-fixing bacterium Azospirillum humicireducens type strain SgZ-5T
Source: Stand Genomic Sci. 2018 Oct 16;13:28. doi: 10.1186/s40793-018-0322-2 (PMC6192227; doi:10.1186/s40793-018-0322-2)
Supplement: Supplementary file 1 — Phylogenetic tree based on the partial nifH gene sequences showing the position of A. humicireducens SgZ-5T relative to other species within the genus Azospirillum and related genus. The strains and their corresponding GenBank accession numbers of nifH gene were indicated in parentheses. The sequences were aligned using Clustal W and the neighbor-joining tree was constructed based on kimura 2-paramenter distance model by using MEGA 5. Bootstrap values above 50% were obtained from 1000 bootstrap replications. Bar, 0.01 substitutions per nucleotide position. Leptospirillum ferriphilum YSKT was used as an outgroup. (DOCX 64 kb) [file 40793_2018_322_MOESM1_ESM.docx]

*Azospirillum oryzae* JCM 21588^T^ (AB594477)

*Azospirillum zeae* Gr61^T^ (FR669147)

*Azospirillum melinis* LMG 24250^T^ (GU256450)

*Azospirillum thiophilum* BV-S^T^ (CP012401)

*Azospirillum doebereinerae* DSM 13131^T^ (FJ799358)

*Azospirillum lipoferum* 4B^T^ (FQ311868)

*Azospirillum picis* DSM 19922^T^ (GU256451)

*Azospirillum humicireducens* SgZ-5^T^ (CP015285)

*Azospirillum* sp. B510 (AP010946)

*Azospirillum brasilense* Sp7^T^ (CP012914)

*Azospirillum rugosum* DSM 19657^T^ (GU256452)

*Azospirillum canadense* LMG 23617^T^ (GU256446)

*Azospirillum halopraeferens* DSM 3675^T^ (GU256447)

*Rhodospirillum centenum* SW^T^ (CP000613)

*Rhodobacter sphaeroides* ATCC 17025^T^ (CP000661)

*Defluviimonas alba* cai42^T^ (CP012661)

*Rhodovulum sulfidophilum* SNK001^T^ (CP015421)

*Sinorhizobium fredii* USDA 257^T^ (CP003565)

*Mesorhizobium loti* NZP2037^T^ (CP016079)

*Mesorhizobium alhagi* CCNWAX44-1^T^ (FJ481880)

*Rhizobium populi* K-38^T^ (KF939630)

*Azorhizobium caulinodans* ORS 571^T^ (AP009384)

*Leptospirillum ferriphilum* YSK^T^ (CP007243)

92

74

83

54

84

81

77

80

79

69

69

50

0.02

**Additional file 1:** Phylogenetic tree based on the partial *nifH* gene sequences showing the position of *A. humicireducens* SgZ-5^T^ relative to other species within the genus *Azospirillum* and related genus. The strains and their corresponding GenBank accession numbers of *nifH* gene were indicated in parentheses. The sequences were aligned using Clustal W and the neighbor-joining tree was constructed based on kimura 2-paramenter distance model by using MEGA 5. Bootstrap values above 50 % were shown obtained from 1000 bootstrap replications. *Bar*, 0.01 substitutions per nucleotide position. *Leptospirillum ferriphilum* YSKT was used as an outgroup.
