# Supplementary material for: Complete genome sequence of the nitrogen-fixing bacterium Azospirillum humicireducens type strain SgZ-5T
Source: Stand Genomic Sci. 2018 Oct 16;13:28. doi: 10.1186/s40793-018-0322-2 (PMC6192227; doi:10.1186/s40793-018-0322-2)
Supplement: Supplementary file 2 — Genes of A. humicireducens SgZ-5T involved in biosynthesis of tryptophan. (DOCX 16 kb) [file 40793_2018_322_MOESM2_ESM.docx]

**Additional file 2:** Genes of *A. humicireducens* SgZ-5^T^ involved in biosynthesis of tryptophan.

| **Locus Tag** | **Size/aa** | **Gene** | **Gene product** |
| --- | --- | --- | --- |
| A6A40_04645 | 804 | *trpC* | Indole-3-glycerol phosphate synthase |
| A6A40_04650 | 1,035 | *trpD* | Anthranilate phosphoribosyltransferase |
| A6A40_04655 | 609 | *trpG* | Anthranilate synthase component II |
| A6A40_04380 | 1,512 | *trpE* | Anthranilate synthase component I |
| A6A40_05775 | 2,247 | *trpEG* | Anthranilate synthase |
