# Supplementary material for: Complete genome sequence of the nitrogen-fixing bacterium Azospirillum humicireducens type strain SgZ-5T
Source: Stand Genomic Sci. 2018 Oct 16;13:28. doi: 10.1186/s40793-018-0322-2 (PMC6192227; doi:10.1186/s40793-018-0322-2)
Supplement: Supplementary file 3 — Genes of A. humicireducens SgZ-5T located in a terpene gene cluster. (DOCX 16 kb) [file 40793_2018_322_MOESM3_ESM.docx]

**Additional file 3:** Genes of *A. humicireducens* SgZ-5^T^ located in a terpene gene cluster.

| **Locus Tag** | **Size/aa** | **Function** |
| --- | --- | --- |
| A6A40_04945 | 1,272 | Serine--tRNA ligase |
| A6A40_04950 | 783 | 5'/3'-nucleotidase SurE |
| A6A40_04955 | 654 | Protein-L-isoaspartate O-methyltransferase |
| A6A40_04960 | 1,266 | Gamma-D-glutamyl-meso-diaminopimelate peptidase |
| A6A40_04965 | 897 | AAA family ATPase |
| A6A40_04970 | 432 | Preprotein translocase subunit YajC |
| A6A40_04975 | 1,599 | Preprotein translocase subunit SecD |
| A6A40_04980 | 957 | Preprotein translocase subunit SecF |
| A6A40_04985 | 378 | Hypothetical protein1 |
| A6A40_04990 | 594 | Superoxide dismutase |
| A6A40_04995 | 1,401 | Phytoene dehydrogenase |
| A6A40_05000 | 933 | Squalene synthase HpnD |
| A6A40_05005 | 873 | Squalene synthase |
| A6A40_05010 | 855 | Phytoene synthase |
| A6A40_05015 | 414 | Glutathione transferase |
| A6A40_05020 | 1,344 | Methylenetetrahydrofolate--tRNA-(uracil(54)-C(5))-methy ltransferase (FADH(2)-oxidizing) TrmFO |
| A6A40_05025 | 408 | Hypothetical protein1 |
| A6A40_05030 | 303 | Hypothetical protein1 |
| A6A40_05035 | 195 | Hypothetical protein1 |
| A6A40_05040 | 2,868 | Excinuclease ABC subunit A |
| A6A40_05045 | 1,605 | Metal-chelation protein CHAD |
| A6A40_05050 | 519 | Phosphohistidine phosphatase |
| A6A40_05055 | 2,148 | RNA degradosome polyphosphate kinase |
